# PAI-1 promoter 4G/5G polymorphism (rs1799768) contributes to tumor susceptibility: Evidence from meta-analysis

**DOI:** 10.3892/etm.2012.734

**Published:** 2012-10-02

**Authors:** XIN XU, YANQI XIE, YIWEI LIN, XIANGLAI XU, YI ZHU, YEQING MAO, ZHENGHUI HU, JIAN WU, HONG CHEN, XIANGYI ZHENG, JIE QIN, LIPING XIE

**Affiliations:** Department of Urology, First Affiliated Hospital, School of Medicine, Zhejiang University, Hangzhou, Zhejiang 310003, P.R. China

**Keywords:** plasminogen activator inhibitor-1, carcinoma, genetic polymorphism, susceptibility, meta-analysis

## Abstract

Plasminogen activator inhibitor-1 (PAI-1), belonging to the urokinase plasminogen activation (uPA) system, is involved in cancer development and progression. The PAI-1 promoter 4G/5G polymorphism was shown to contribute to genetic susceptibility to cancer, although the results were inconsistent. To assess this relationship more precisely, a meta-analysis was performed. The electronic databases PubMed, Scopus, Web of Science and Chinese National Knowledge Infrastructure (CNKI) were searched; data were extracted and analyzed independently by two reviewers. Ultimately, 21 eligible case-control studies with a total of 8,415 cancer cases and 9,208 controls were included. The overall odds ratio (OR) with its 95% confidence interval (CI) showed a statistically significant association between the PAI-1 promoter 4G/5G polymorphism and cancer risk (4G/4G vs. 5G/5G: OR=1.25, 95% CI=1.07–1.47, P_heterogeneity_=0.001; 4G/4G vs. 4G/5G+5G/5G: OR=1.10, 95% CI=1.03–1.17, P_heterogeneity_=0.194; 4G/4G+4G/5G vs. 5G/5G: OR=1.17, 95% CI=1.01–1.35, P_heterogeneity_=0.041). In further subgroup analyses, the increased risk of cancer was observed in a subgroup of Caucasians with regards to endometrial cancer. Our meta-analysis suggests that the PAI-1 4G/5G polymorphism most likely contributes to susceptibility to cancer, particularly in Caucasians. Furthermore, the 4G allele may be associated with an increased risk of endometrial cancer.

## Introduction

Despite many years of primary and clinical research aimed at curbing tumor growth, metastasis remains the main cause of mortality in cancer patients (1). During recent years, mounting evidence has shown that plasminogen activator inhibitor-1 (PAI-1), belonging to the urokinase plasminogen activation (uPA) system, plays an important role in signal transduction, cell adherence and cell migration, thus promoting invasion and metastasis (2). In addition, PAI-1 concentrations and mRNA levels in primary tumor tissues correlate with adverse patient outcome in multiple cancer types (3–6). Recently, the first level-of-evidence-1 (LOE-1) cancer biomarker, PAI-1, entered clinical practice in breast cancer management (7).

The PAI-1 gene is located on chromosome 7 and contains 8 introns and 9 exons (8). Gene variability may contribute to the level of PAI-1 biosynthesis. Among the variants of the PAI-1 gene, the PAI-1 4G/5G polymorphism (rs1799768) has been the most frequently studied. The 4G allele of 4G/5G insertion/deletion polymorphism located in the promoter region 675 bp upstream from the transcription start sequence of the PAI-1 gene is responsible for higher plasma PAI-1 levels, conditioning a clear hypofibrinolytic state (9). Several studies (10,11) have shown that the 4G allele has greater activity than the 5G allele and that higher frequencies of the 4G allele are associated with elevated plasma levels of PAI-1.

To date, a number of molecular epidemiological studies have been performed to evaluate the association between PAI-1 promoter 4G/5G polymorphism and risks for different types of tumor, including breast cancer (12–17), colorectal cancer (3,18–20), ovarian cancer (21,22), oral cancer (23,24), endometrial cancer (25,26) and other cancers (27,28), in diverse populations However, the observed associations of these studies were inconsistent and a single study may be insufficient to detect a possible small effect of the polymorphism on cancer, particularly when the sample size is relatively small. Hence, we performed a meta-analysis of all eligible studies to derive a more precise estimation of the association of PAI-1 promoter 4G/5G polymorphism with cancer risk. To our knowledge, no meta-analysis concerning the influence of PAI-1 4G/5G polymorphism on cancer risk has been published in the literature.

## Materials and methods

### Publication search

We carried out a search in PubMed, Scopus, Web of Science and Chinese National Knowledge Infrastructure (CNKI) databases with a combination of the following keywords: ‘plasminogen activator inhibitor-1’, ‘PAI-1’, ‘SERPINE1’, ‘polymorphism’, ‘variation’, ‘variant’, ‘carcinoma’, ‘tumor’ and ‘cancer’ (the last search was updated on 10th February 2012). We evaluated potentially relevant publications by examining their titles and abstracts. All studies with full text matching the eligible criteria were retrieved. Additional relevant studies were identified by a manual search of the references of retrieved articles and reviews.

### Inclusion criteria

Studies included in this meta-analysis met the following criteria: a) evaluation of the link between PAI-1 promoter 4G/5G polymorphism and cancer risk, b) a case-control design, c) contained sufficient published data for estimating odds ratios (ORs) and 95% confidence intervals (CIs).

### Quality assessment

The quality of each study was assessed independently by two reviewers who used the Newcastle-Ottawa Scale (NOS) (29). The NOS consists of three parameters of quality: selection, comparability, and exposure (case-control studies) or outcome (cohort studies). Scores ranged from 0 stars (worst) to 9 stars (best). Studies with a score ≥7 stars were considered to be of high quality. Any discrepancies were settled by a joint re-evaluation of the original article with a third reviewer.

### Data extraction

Information was extracted independently by two reviewers according to the inclusion criteria. Any disagreement was settled by consensus among the two. For each study, the following characteristics were collected: the first author’s last name, year of publication, country of origin, ethnicity, numbers of genotyped cases and controls, source of control groups and genotyping methods. Different ethnic descents were categorized as Caucasian, Asian and mixed, which included more than one ethnic descent. We also contacted study authors for any missing data.

When the source of controls was not clearly defined in the paper, it was defined ‘not specified’ to be conservative. The ethnicity of each study population was defined as the ethnic group of ≥90% of the study subjects. When the paper did not specify the ethnicity of the study population, it was hypothesized based on the most frequent ethnic group in the study country.

### Statistical methods

Crude ORs together with their corresponding 95% CIs were used to calculate and assess the strength of association between PAI-1 promoter 4G/5G polymorphism and cancer risk. We first estimated cancer risk associated with PAI-1 promoter 4G/5G polymorphism by 4G/4G and 4G/5G genotypes compared with the 5G/5G (co-dominant model) and then evaluated 4G/4G+4G/5G vs. 5G/5G, and 4G/4G vs. 4G/5G+5G/5G, assuming the dominant and recessive effects of the variant 4G allele, respectively. The departure of frequencies from those expected under Hardy-Weinberg equilibrium (HWE) was assessed by Chi-square goodness-of-fit tests in controls. Stratified analyses were also performed by cancer type (if a cancer type was represented by only a single study, it was combined with the ‘other cancers’ group) and ethnicity.

For the heterogeneity test, a random-effect model (the DerSimonian and Laird method) (30) was used when P<0.05, otherwise a fixed-effect model (the Mantel-Haenszel method) (31) was used. In the random-effect model, we incorporated the random variation within the studies and the variation among the different studies, and in the fixed-effect model, we assumed that all studies came from a common population and the effect size was not significantly different among the different studies.

With regard to sensitive analyses, the influence of each study in the pooled OR was examined by repeating meta-analyses while omitting each study one at a time. We performed visual inspection of funnel plots to examine the underlying publication bias, and also used Egger’s weighted regression method to calculate P for bias (32). All statistical analyses were performed with STATA 11.0 (StataCorp, TX, USA), using two-sided P-values.

## Results

### Summary statistics

We identified 21 articles (3,12–28,33–35) which we used to evaluate the association of PAI-1 promoter 4G/5G polymorphism with risk of cancer. The study characteristics are summarized in Table I. Among the 21 eligible case-control studies, which included 8,415 cases and 9,208 controls, there were 7 breast cancer studies, 4 colorectal cancer studies, 3 ovarian cancer studies, 2 oral cancer studies, 2 endometrial cancer studies, and the others were categorized into the ‘other cancer’ group. Continental origin populations among these studies were as follows: 13 studies with Caucasian subjects, 7 studies with Asian subjects, and one study with more than one ethnicity (classified as mixed). Cancers were confirmed histologically in most studies. Diverse genotyping methods were used, including polymerase chain reaction-restriction fragment length polymorphism (PCR-RFLP), polymerase chain reaction-single strand conformation polymorphism (PCR-SSCP), TaqMan, sequencing, minisequencing, real-time PCR and matrix-assisted laser desorption/ionizing time-of-flight mass spectrometry (MALDI/TOF). The distribution of genotypes in the controls was consistent with HWE in all but one of the studies (35).

### Quality assessment results

The NOS results showed that 81.0% of the studies were of high quality (NOS score >6), with an average NOS score of 7.6, which indicated that the methodological quality was generally good. We defined studies that scored ≥7 as having high methodological quality, and we judged 4 (21,22,26,35) of the 21 studies to be of low quality (score of 6) primarily due to the absence of a definition of controls and a lack of a description of comparability between cases and controls.

### Quantitative synthesis

**Figure 1:**
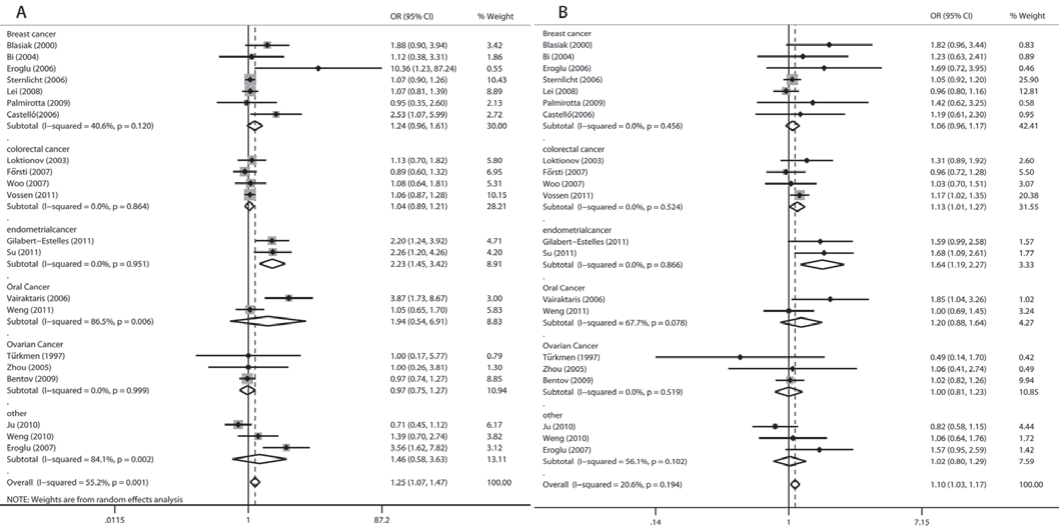
Forest plot of cancer risk associated with plasminogen activator inhibitor-1 (PAI-1) 4G/5G polymorphism in different ethnicities. The squares and horizontal lines correspond to the study-specific odds ratio (OR) and 95% confidence interval (CI). The area of the squares reflects the study-specific weight (inverse of the variance). The diamond represents the pooled OR and 95% CI. (A) 4G/4G vs. 5G/5G, random-effect model used as the P-value for the heterogeneity test <0.05; (B) 4G/4G vs. 4G/5G+5G/5G.

**Figure 2:**
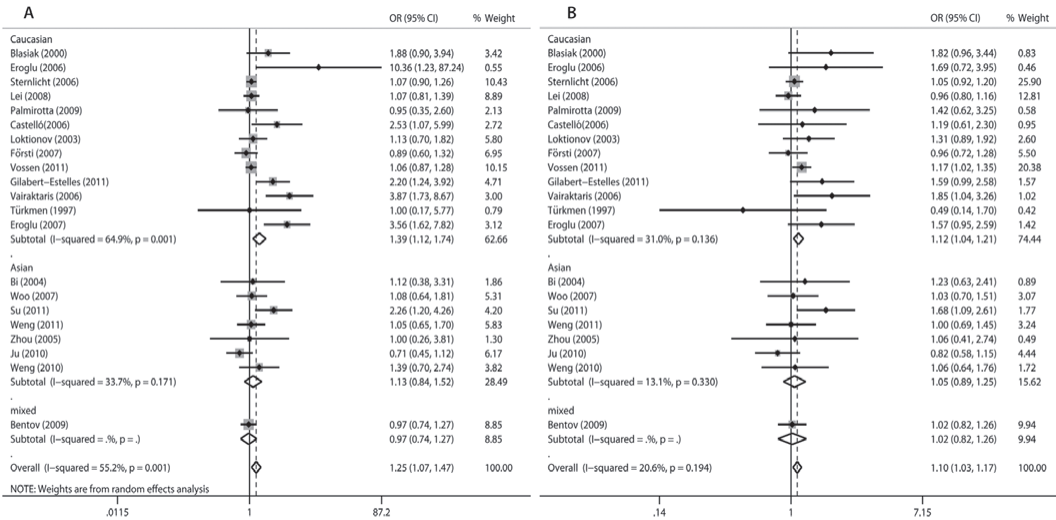
Forest plot of cancer risk associated with plasminogen activator inhibitor-1 (PAI-1) 4G/5G polymorphism in different types of cancer. The squares and horizontal lines correspond to the study-specific odds ratio (OR) and 95% confidence interval (CI). The area of the squares reflects the study-specific weight (inverse of the variance). The diamond represents the pooled OR and 95% CI. (A) 4G/4G vs. 5G/5G, random-effect model used as the P-value for the heterogeneity test <0.05; (B) 4G/4G vs. (4G/5G+5G/5G).

The overall OR with its 95% CI revealed a statistical association between the PAI-1 promoter 4G/5G polymorphism and the risk of cancer (4G/4G vs. 5G/5G: OR=1.25, 95% CI=1.07–1.47, P_heterogeneity_=0.001; 4G/4G vs. 4G/5G+5G/5G: OR=1.10, 95% CI=1.03–1.17, P_heterogeneity_=0.194; 4G/4G+4G/5G vs. 5G/5G: OR=1.17, 95% CI=1.01–1.35, P_heterogeneity_=0.041; Figs. 1 and 2). In the subgroup analysis by ethnicity, statistically significant elevated cancer risks were found among Caucasians (4G/4G vs. 5G/5G: OR=1.39, 95% CI=1.12–1.74, P_heterogeneity_=0.001; 4G/4G vs. 4G/5G+5G/5G: OR=1.12, 95% CI=1.04–1.21, P_heterogeneity_=0.136; 4G/4G+4G/5G vs. 5G/5G: OR=1.28, 95% CI=1.04–1.57, P_heterogeneity_=0.000; Fig. 1). Moreover, when stratifying by cancer type, significantly increased cancer risks were observed for endometrial cancer in all comparison models (4G/4G vs. 5G/5G: OR=2.23, 95% CI=1.46–3.42, P_heterogeneity_=0.951; 4G/5G vs. 5G/5G: OR=1.56, 95% CI=1.08–2.25, P_heterogeneity_=0.830; 4G/4G vs. 4G/5G+5G/5G: OR=1.64, 95% CI=1.19–2.27, P_heterogeneity_=0.866; 4G/4G+4G/5G vs. 5G/5G: OR=1.74, 95% CI=1.23–2.47, P_heterogeneity_=0.979; Fig. 2). The details are listed in Table II.

### Sensitivity analysis

**Figure 3:**
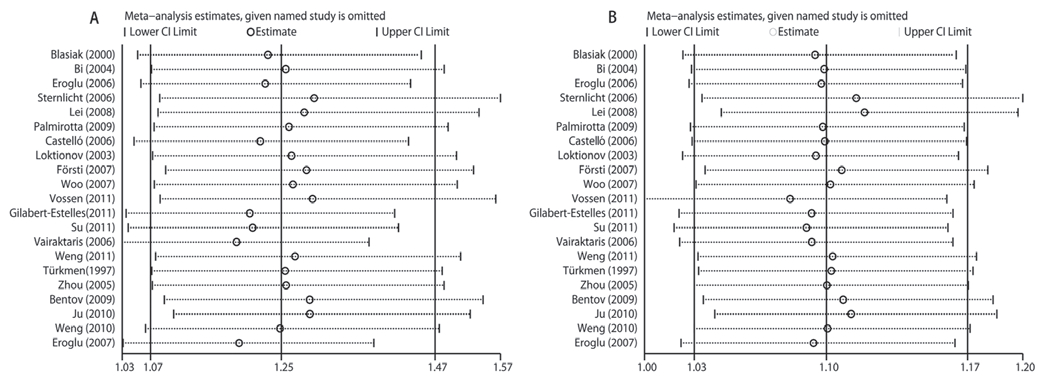
Influence analysis in the overall meta-analysis. The figure shows the influence of individual studies on the summary odds ratio (OR). (A) 4G/4G vs. 5G/5G; (B) 4G/4G vs. 4G/5G+5G/5G. CI, confidence interval.

In the sensitivity analysis (Fig. 3), the influence of each study on the pooled OR was examined by repeating the meta-analysis while omitting each study, one at a time. This procedure demonstrated that our results were reliable and robust. In addition, when excluding the studies that were not in HWE, the estimated pooled OR still did not change at all.

### Publication bias

Publication bias was assessed by visual inspection of funnel plots in which the standard error of the log (OR) of each study was plotted against its log (OR). An asymmetric plot suggests a possible publication bias. Funnel plot asymmetry was assessed by the method of Egger’s linear regression test, a linear regression approach for measuring funnel plot asymmetry on the natural logarithm scale of the OR. The significance of the intercept was determined by the t-test (P<0.05 was considered representative of statistically significant publication bias) (32). As a result, publication bias was identified in certain comparisons (4G/4G vs. 5G/5G, 4G/5G vs. 5G/5G, and 4G/4G+4G/5G vs. 5G/5G).

## Discussion

The promoter 4G/5G polymorphism is the most well-characterized PAI-1 polymorphism, but the reported associations with cancer risk among studies are inconsistent. Our present meta-analysis, incorporating 21 case-control studies (8,415 cases and 9,208 controls), suggests that the promoter 4G/5G polymorphism is significantly associated with increased cancer risk, particularly in Caucasians.

How a promoter 4G/5G polymorphism increases the risk of developing cancer is yet to be established; however, it is known that the transcription of the PAI-1 gene is affected by the presence of a guanosine insertion (5G)/deletion (4G) polymorphism at the promoter region (13,36). Both 4G and 5G alleles bind a transcriptional activator, whereas the 5G allele also binds a repressor (36). Hence, the presence of 4G/4G homozygotes enhances transcription to increase plasma PAI-1 levels, whereas 5G/5G homozygotes are associated with lower levels of the inhibitor (13,36). High plasma PAI-1 concentrations have been reported to be a predictive indicator of poor prognosis and shorter survival of patients with cancer (4–6,37). The PAI-1 4G/5G polymorphism has also been found to be located within the functional binding sites for the transcription factors Smad and NF-κB, which mediate the effects of two known inducers of PAI-1 expression, transforming growth factor-β (TGF-β) and tissue necrosis factor-α (TNF-α), respectively (38,39).

Since tumor origin may influence the results of the meta-analyses, we performed subgroup analyses by cancer type. The association of the PAI-1 promoter 4G/5G polymorphism with increased risk of cancer in this meta-analysis was significant in the endometrial cancer subgroup. In addition to the above reasons, the presence of the 4G allele results in a higher PAI-1 transcription response to cytokines or growth factors than the 5G allele, so the 4G/5G polymorphism may influence tissue PAI-1 levels in endometrial cancer patients through the action of cytokines released by tumor cells (25). However, since only two independent studies with limited sample size were included in this meta-analysis, further studies with a larger number of patients are needed to explore the clinical significance of the PAI-1 4G/5G polymorphism in endometrial cancer.

Due to differences in the underlying genetic backgrounds and/or environmental and social factors of the different populations studied, the incidence of gene polymorphisms varies substantially between different racial or ethnic populations. In this meta-analysis, when stratifying by ethnicity we found that the association between PAI-1 promoter 4G/5G polymorphism and increased risk of cancer was significant only in Caucasians, not in the Asian population. As a multi-factorial disease, the factors causing cancer vary in different populations. Although the reasons for this difference remain controversial, there are several studies showing that it depends on a combination of differences in polymorphism distributions with nongenetic factors. Therefore, the functional difference of this polymorphism of PAI-1 in Caucasians and Asians may result from interactions with environmental and social factors. In addition, considering the multistage character of cancer, genetic factors may play a role at specific stages only, which may vary between populations. Therefore, larger-scale studies and combined analysis are warranted to further confirm the effect of ethnic difference in this polymorphism on cancer risks. These studies should be stratified by ethnicity, differentiated by the stage of carcinogenesis and include an assessment of interactions with environmental factors.

Additionally, heterogeneity may influence the results of meta-analyses. Evident heterogeneity between studies was observed in overall comparisons and also in certain subgroup analyses. Through the subgroup analysis, we found that the source of heterogeneity was from types of cancer and ethnicity, suggesting that tumor site and population play an important role for the same polymorphism.

When interpreting the results, the following limitations of the study should be considered. First, only published articles were included in this study. Although we conducted a thorough and comprehensive search and also contacted study authors for missing data (13), publication bias could not be completely avoided. To evaluate the results, Egger’s test was performed. The results show that key publication bias may be present in this meta-analysis as there was some uncertainty with the P-value being less than 0.05 in Egger’s test, hence some results should be interpreted cautiously. Secondly, lack of the original data of the reviewed studies limited our further evaluation of potential interactions, since the interactions between gene-to-gene, gene-to-environment and even different polymorphic loci of the same gene may modulate cancer risk.

Our results suggest that the PAI-1 promoter 4G/5G polymorphism is a potential clinical genetic marker contributing to cancer susceptibility. Whether it could be applied to genotyping for clinical assessment requires large-scale population studies among different ethnicities and regions.

## Figures and Tables

**Table I:** Characteristics of populations and cancer types of studies included in the meta-analysis.

Study (year)	Country of origin	Ethnicity	Source of control	Cancer type	Genotyping method	Cases (no.)	Controls (no.)	HWE
Türkmen 1997	German	Caucasian	ns	Ovarian	PCR-RFLP	22	23	Yes
Blasiak 2000	Poland	Caucasian	ns	Breast	PCR-SSCP	100	106	Yes
Loktionov 2003	UK	Caucasian	PCC	colorectal	PCR-SSCP	206	355	Yes
Bi 2004	China	Asian	PCC	Breast	PCR-SSCP	53	146	No
Zhou 2005	China	Asian	HCC	Ovarian	PCR-SSCP	52	30	Yes
Eroglu 2006	Turkey	Caucasian	ns	Breast	PCR-SSCP	34	90	Yes
Castellò 2006	Spain	Caucasian	PCC	Breast	PCR-SSCP	104	104	Yes
Sternlicht 2006	USA	Caucasian	PCC	Breast	Minisequencing	2,539	1,832	Yes
Vairaktaris 2006	Greece	Caucasian	PCC	Oral	PCR-RFLP	104	106	Yes
Eroglu 2007	Turkey	Caucasian	ns	Mixed	PCR–SSCP	125	180	Yes
Försti 2007	German	Caucasian	PCC	Colorectal	Taqman	304	581	Yes
Woo 2007	Korea	Asian	ns	Colorectal	PCR-RFLP	185	304	Yes
Lei 2008	German	Caucasian	PCC	Breast	Taqman	959	952	Yes
Bentov 2009	Canada	Mixed	HCC	Ovarian	MALDI-TOF	775	889	Yes
Palmirotta 2009	Italy	Caucasian	PCC	Breast	Sequencing	99	50	Yes
Ju 2010	Korea	Asian	PCC	Gastric	MALDI-TOF	252	406	Yes
Weng 2010	Taiwan	Asian	HCC	Hepatocellular	PCR-RFLP	102	344	Yes
Gilabert-Estelles 2011	Spain	Caucasian	PCC	Endometrial	Real-time PCR	212	211	Yes
Su 2011	Taiwan	Asian	HCC	Endometrial	PCR-RFLP	134	302	Yes
Vossen 2011	Netherlands	Caucasian	PCC	Colorectal	Taqman	1,801	1,853	Yes
Weng 2011	Taiwan	Asian	HCC	Oral	PCR-RFLP	253	344	Yes

HCC, hospital-based case-control; PCC, population-based case-control; ns, not specified (source of controls was not clearly defined); PCR-RFLP, polymerase chain reaction-restriction fragment length polymorphism; PCR-SSCP, single-strand conformation polymorphism analysis of polymerase chain reaction products; MALDI-TOF, matrix-assisted laser desorption/ionizing time-of-flight mass spectrometry; HWE, Hardy-Weinberg equilibrium.

**Table II:** Stratified analyses of the plasminogen activator inhibitor1 (PAI-1) 4G/5G polymorphism and cancer risk.

		4G/4G vs. 5G/5G		4G/5G vs. 5G/5G		Dominant model		Recessive model	
Variables	No. of studies (cases/controls)	OR (95% CI)	P-value^a^	OR (95% CI)	P-value^a^	OR (95% CI)	P-value^a^	OR (95% CI)	P-value^a^
Total	21 (8415/9208)	**1.25 (1.07–1.47)**	0.001	1.12(0.97–1.30)	0.002	**1.17 (1.01–1.35)**	0.041	**1.10 (1.03–1.17)**	0.194
Cancer type									
Breast	7 (3888/3280)	1.13 (0.99–1.30)	0.120	1.15 (0.86–1.54)	0.032	1.19 (0.91–1.56)	0.272	1.06 (0.96–1.17)	0.456
Colorectal	4 (2496/3093)	1.04 (0.89–1.21)	0.864	0.88 (0.76–1.01)	0.852	0.93 (0.82–1.07)	0.941	**1.13 (1.01–1.27)**	0.524
Oral	2 (357/450)	1.94 (0.54–6.91)	0.006	1.67 (0.63–4.41)	0.028	1.80 (0.60–5.45)	0.008	1.20 (0.88–1.64)	0.078
Endometrial	2 (346/513)	**2.23 (1.46–3.42)**	0.951	**1.56 (1.08–2.25)**	0.830	**1.74 (1.23–2.47)**	0.979	**1.64 (1.19–2.27)**	0.866
Ovarian	3 (849/942)	0.97 (0.75–1.27)	0.999	0.95 (0.75–1.21)	0.469	0.96 (0.77–1.20)	0.746	1.00 (0.81–1.23)	0.519
Other	3 (479/930)	1.46 (0.58–3.63)	0.002	1.42 (0.71–2.83)	0.013	1.43 (0.66–3.09)	0.003	1.02 (0.80–1.29)	0.102
Ethnicity									
Caucasian	13 (6609/6443)	**1.39 (1.12–1.74)**	0.001	**1.21 (0.98–1.50)**	0.000	**1.28 (1.04–1.57)**	0.000	**1.12 (1.04–1.21)**	0.136
Asian	7 (1031/1876)	1.10 (0.88–1.38)	0.171	1.07 (0.87–1.32)	0.718	1.08 (0.89–1.32)	0.393	1.05 (0.89–1.25)	0.330

OR, odds ratio; CI, confidence interval.

a P-value of Q-test for heterogeneity. Random-effects model was used when P-value for heterogeneity test <0.05; otherwise, fixed-effects model was used. Bold values indicate a significant difference.
